# Risk Profiles of Older Rural Residents With Functional, Nutritional, and Social Needs

**DOI:** 10.1093/geroni/igaa057.124

**Published:** 2020-12-16

**Authors:** Raven Weaver

**Affiliations:** Washington State University, Pullman, Washington, United States

## Abstract

Community-based service organizations are well positioned to address social determinants of health by offering a range of services/supports to community residents. To identify health needs and service delivery gaps among a geographically and economically diverse eight-county region, a needs assessment was conducted to support community-based agencies efforts to better support aging residents. A random sample of adults responded to the survey, with 1,280 respondents aged 60+ (mean age=71); the majority of participants were White, female, retired, reported at least some college education, and lived with at least one person. Cluster analysis distinguished three groups of residents, informed by typical enrollment-type data and a social engagement index. A series of one-way ANCOVA and chi-square analyses were conducted to examine how low-, moderate-, and high-risk groups differed on social, nutritional, and functional health needs. High-risk respondents were significantly more likely to report needing social, nutritional, and functional health services, compared to moderate- and low-risk respondents. High-risk respondents were more likely to experience barriers to seeing a physician (X2=34.054, p<.001), a non-emergency ED visit (X2=22.799, p<.001), and an unplanned hospital visit (X2=14.484, p=.001) compared to members of either low- or moderate-risk groups. Ongoing efforts to identify high-risk residents and proactively target moderate-risk residents support low-cost community interventions (i.e., assessing residents for services in locations regularly attended, such as senior meal centers), rather than high-cost interventions (e.g., emergency care, hospitalizations) are essential. Findings inform community-based outreach approaches that target social, economic, and environmental factors essential in improving health and achieving health equity.

